# Benefit of a single simulated hypobaric hypoxia in healthy mice performance and analysis of mitochondria-related gene changes

**DOI:** 10.1038/s41598-020-80425-8

**Published:** 2021-02-24

**Authors:** Fei-Fei Wu, Kun-Long Zhang, Zheng-Mei Wang, Yi Yang, Shao-Hua Li, Jia-Qi Wang, Jin Ma, Yan-Ling Yang, Hai-Feng Zhang, Ya-Yun Wang

**Affiliations:** 1 Specific Lab for Mitochondrial Plasticity Underlying Nervous System Diseases, National Demonstration Center for Experimental Preclinical Medicine Education, Air Force Medical University, Xi’an, 710032 China; 2 Department of Rehabilitation and Physical Therapy, Xi-Jing Hospital, Air Force Medical University, Xi’an, 710032 China; 3 Department of Aerospace Physiology, Air Force Medical University, Xi’an, 710032 China; 4 Department of Hepatobiliary Surgery, Xi-Jing Hospital, Air Force Medical University, Xi’an, 710032 China

**Keywords:** Neuroscience, Medical research

## Abstract

Simulated hypobaric hypoxia (SHH) training has been used to enhance running performance. However, no studies have evaluated the effects of a single SHH exposure on healthy mice performance and analyzed the changes of mitochondria-related genes in the central nervous system. The current study used a mouse decompression chamber to simulate mild hypobaric hypoxia at the high altitude of 5000 m or severe hypobaric hypoxia at 8000 m for 16 h (*SHH5000* & *SHH8000*, respectively). Then, the mouse behavioral tests were recorded by a modified Noldus video tracking. Third, the effects of SHH on 8 mitochondria-related genes of Drp1, Mfn1, Mfn2, Opa1, TFAM, SGK1, UCP2 and UCP4, were assessed in cerebellum, hippocampus and gastrocnemius muscles. The results have shown that a single mild or severe HH improves healthy mice performance. In cerebellum, 6 of all 8 detected genes (except Mfn2 and UCP4) did not change after SHH. In hippocampus, all detected genes did not change after SHH. In muscles, 7 of all 8 detected genes (except Opa1) did not change after SHH. The present study has indicated the benefit of a single SHH in healthy mice performance, which would due to the stabilized mitochondria against a mild stress state.

## Introduction

Hypoxia is defined as oxygen deprivation at the tissue level^1^. High altitude causes hypoxia due to decreased inspired oxygen pressure, which is called hypobaric hypoxia (HH)^2^. This atmospheric pressure is composed of the partial pressures of the constituent gases, oxygen, nitrogen, and water vapor (6.3 kPa at 37 ℃). As oxygen accounts for 21% of the dry air, the formula of inspired oxygen pressure is 0.21 '(100 − 6.3) = 19.6 kPa at sea level^3^. Atmospheric pressure and inspired oxygen pressure decrease linearly with altitude. The inspired oxygen pressure at 5000 m or at 8000 m decreases to be 56.2% or 38.3% of the sea level value, respectively^3^. Hence at 5000 m or 8000 m above sea level, the inspired oxygen pressure decreases to 19.6 × 0.562 = 11.01 kPa or 19.6 × 0.383 = 7.51 kPa, respectively.

Many previous results have indicated that HH could induce neurological damages, including high-altitude headache, acute mountain sickness, and high-altitude cerebral edema^4^. HH could result in impairment in arithmetic^5^ memory and metamemory language^6^, perception, learning, cognitive flexibility, and psychomotor skills^7^. On the contrary, HH training has been used by endurance athletes in the pursuit of performance enhancement after returning to sea level. Short-term “live high” training will increase O_2_ transport and delay hypoxic ventilatory responses, enhancing the 3000/5000 m run-time trial time^8^. The use of HH or simulated hypobaric hypoxia (SHH) has become increasingly popular sincerity was discussed in the first international symposium on altitude training and team sports held in 2013^9^. The beneficial effects of HH or SHH on athletes have focused on skeletal tissues and the cardiac system, such as hypobaric hypoxic exercise superimposed on hypoxic residence, which could boost the skeletal muscle mitochondrial transcriptional response in elite port athletes^10^. However, it should be noted that the neurological physiological responses under HH remain debated.

A fall in inspired oxygen pressure reduces the driving pressure for gas exchange in the lungs and in turn produces a cascade of effects right down to the level of the mitochondria, the final destination of the oxygen^11^. In the central nervous system, mitochondria are involved in neuronal impairment induced by HH. It has been shown that dihydromyricetin improved mitochondrial function and reduced oxidative stress under HH^12^. Mironova GD et al. found that cortical mitochondria responded to HHconditions with typical and progressive changes in the structure and function of mitochondrial enzymes^13^. Therefore, the capability of mitochondrial stress is closely related to HH-induced stress.

The mitochondria-directed interventions affecting brain susceptibility to injury including mitochondrial dynamics, mitochondrial biogenesis, mitophagy, and morphogenesis. The mitochondrial fusion–fission cycle is important for the maintenance of organelle function. The process of mitochondrial fission is driven by dynamin-related protein 1 (Drp1), a cytosolic protein that upon activation translocates to the outer mitochondrial membrane^14^*.* Fusion depends on mitofusin 1 (Mfn1) and mitofusin 2 (Mfn2) in the outer mitochondrial membranes^15^. Optic atrophy 1 (Opa1) mediates mitochondrial inner membrane fusion^16^. Mitochondrial biogenesis is a highly regulated process, and mitochondrial transcription factor A (TFAM) is needed^17^. Mitophagy is an important mechanism controlling mitochondrial quality, and serum and glucocorticoid-regulated protein kinase 1 (SGK1) induces the autophagic degradation of mitochondria^18^. Mitochondrial uncoupling proteins (UCPs) are present in neurons, and UCP2 & UCP4can directly influence neurotransmission, synaptic plasticity and neuronal processes by regulating mitochondrial biogenesis, calcium flux, and free radical production^19^.

In the current study, we first used a mouse decompression chamber to simulate mild hypobaric hypoxia at the high altitude of 5000 m or severe hypobaric hypoxia at 8000 m for 16 h, which are named as SHH*5000* & SHH*8000* in the present study, respectively. Then, the mouse behavioral tests were recorded by a modified Noldus video tracking. Third, the effects of hypobaric hypoxia on 8 mitochondria-related genes of Drp1, Mfn1, Mfn2, Opa1, TFAM, SGK1, UCP2 and UCP4, were assessed in cerebellum, hippocampus and gastrocnemius muscle. The aim of the present work was to describe the changes in mitochondrial dynamics in the central nervous system of healthy mice caused by exposure to a single hypobaric hypoxia event.

## Results

### A single SHH improves healthy mice performance detected by a modified Noldus video tracking

**Figure 1:**
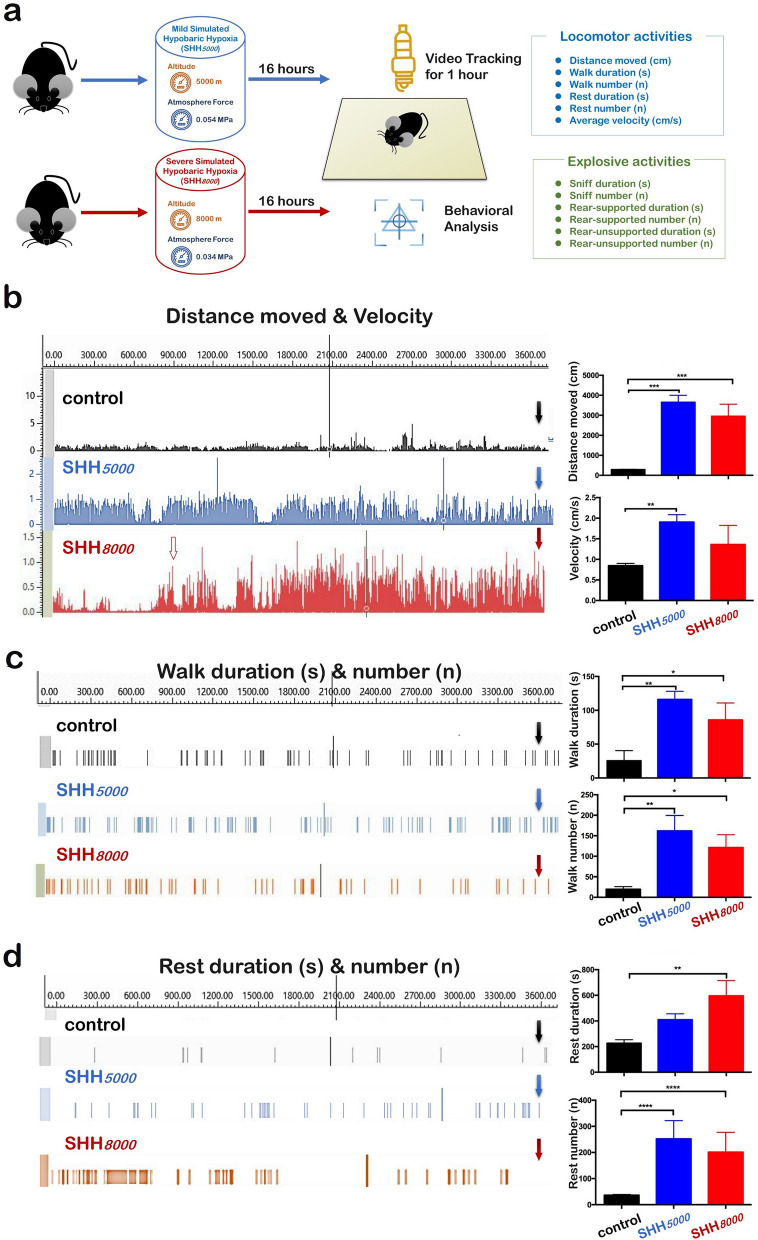

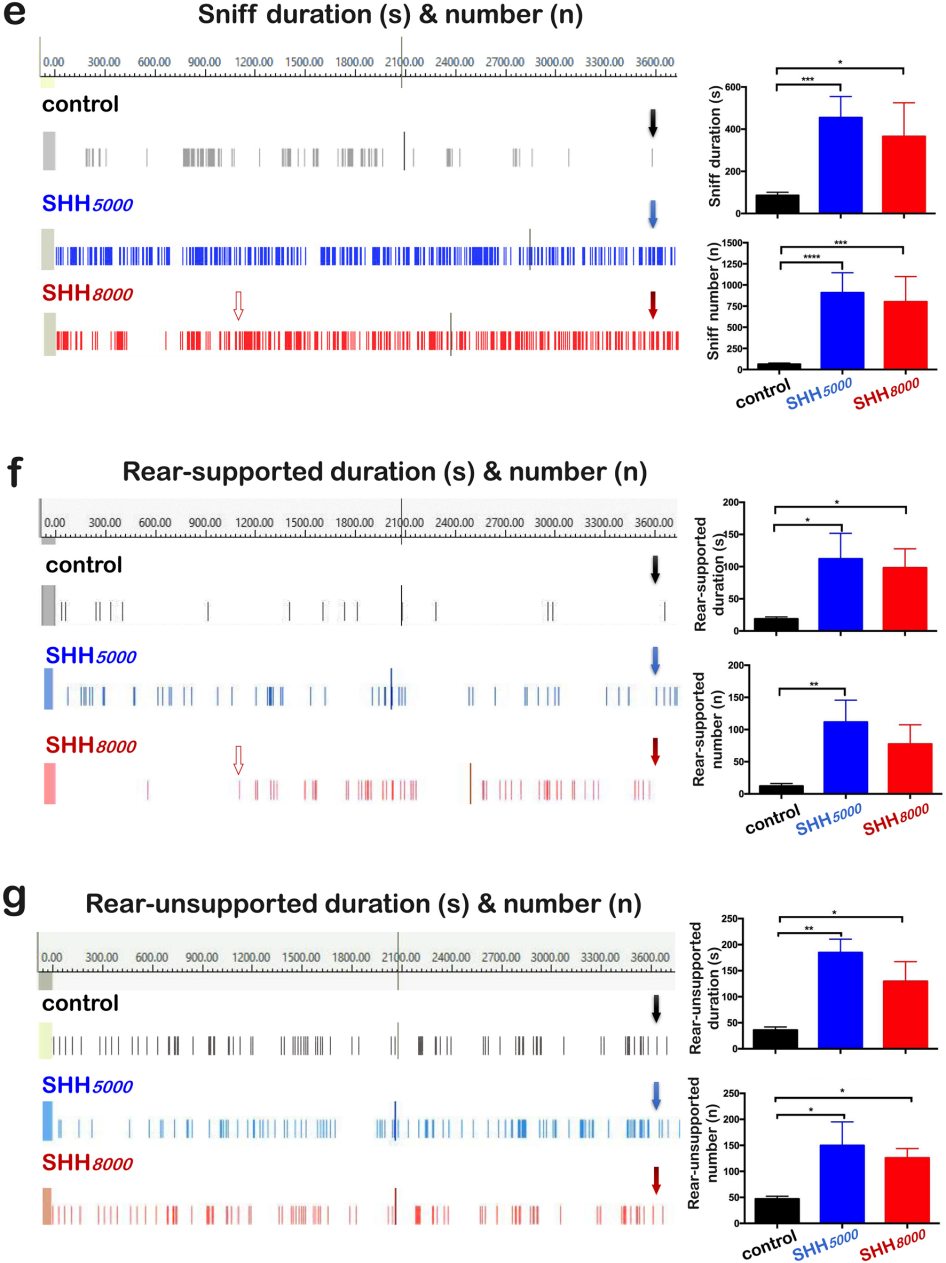
Locomotor activity and exploratory behavior were improved by a short-term simulated hypobaric hypoxia in healthy mice. (**a**) Scheme of 3600 s of video tracking and behavioral analysis after 16 h of SHH*5000* or SHH*8000* treatment, aimed to examine the cerebellum susceptibility to hypoxia. Most locomotor activities [(**b**) distance moved and velocity; (**c**) walk duration and walk number; (**d**) Rest duration and rest number] were significantly increased by SHH*5000*and SHH*8000*treatment. Several exploratory behaviors [(**e**) sniff duration and sniff number; (**f**) rear-supported duration and sniff number; (**g**) rear-unsupported duration] were increased by SHH*5000* and SHH*8000* treatment. The left column in each figure from B to G shows the representative ground truth and generated annotation over time (3600 s) for test video tracking; the solid arrow represents the time point of termination for statistical analysis (3600 s); the hollow arrow represents the time point for changing behavioral activity in the SHH*8000* group. The right column in each figure from B to G shows the comparison of the means of behavior activities over the 3600 s for the treated animals (SHH*5000* or SHH*8000*) and their respective control. Each value is expressed as the mean ± standard error of the mean. Multiple comparisons between groups were carried out with Tukey’s post hoc test. *P value < 0.05; **P value < 0.01; ***P value < 0.001, n = 10 per group.

As above mentioned research revealed, the athletes short-term “live high” training will increase in O_2_ transport and delay hypoxic ventilatory responses, enhancing 30005000 m run-time trial time^8^. Therefore, we tested whether single 16 h exposure to mild environmental hypoxia (breathing the oxygen with the decreased pressure from 19.6 kPa to 11.01 kPa and equivalent to 5000 m altitude) or the severe environmental hypoxia (breathing the oxygen with the decreased pressure 7.51 kPa and equivalent to 8000 m altitude) could improve behavioral performance on healthy mice. Scheme of 3600 s of video tracking and behavioral analysis was shown in Fig. 1a.

First, we analyzed spontaneous locomotor activities. Laboratory studies showed a total distance moved of 286.5 ± 8.1 cm in normal mice (n = 10), as shown in Fig. 1b. SHH*5000* and SHH*8000* significantly increased distance traveled to 12.7 times (*P* = 0.0002) and 10.3 times (*P* = 0.0008) as much as that of normal mice, respectively. It should be noted that distance moved began to increase only 500 s after SHH*8000*, indicated by the hollow arrow representing the time point for changing in Fig. 1b. And there was no difference between the increase by SHH*5000* and by SHH*8000.*

In Fig. 1b and supplementary Video 1, laboratory studies showed average velocity of 0.8 ± 0.03 cm/s in normal mice. SHH*5000* significantly increased average velocity to 2.4 times as much as that of normal mice (Supplementary Video 2) (*P* = 0.0099). SHH*8000* average velocity of to 1.7 times as much as that of normal mice, but there was no difference between control versus SHH*8000.*

In Fig. 1c, the video tracking studies showed a total walk duration of 25.4 ± 8.7 s and walk number of 19.7 ± 3.7 in normal mice. SHH*5000* and SHH*8000* significantly increased walk duration to 4.6 times and 3.4 times, as well as 8.2 times and 6.2 times as much as that of normal mice, respectively.

Next, we analyzed spontaneous rest states. As shown in Fig. 1d and Supplementary Video 1, laboratory studies showed a total rest duration of 226.4 ± 15.9 s in normal mice. SHH*5000* increased rest duration to 1.8 times as much as that of normal mice (Supplementary Video 2), but there was no significance between control and SHH*5000* (*P* = 0.0558). Interestingly, SHH*8000* significantly increased rest duration to 2.6 times as much as that of normal mice (Supplementary Video 3) (*P* = 0.0023). It was logical to observe a significant increase of average velocity in SHH*5000* group, but no statistical difference between the control and SHH*8000* groups in the average velocity.

Then we assessed the exploratory behavior of hypobaric hypoxia at altitude 5000 m or 8000 m using a battery of behavioral tests.

As shown in Fig. 1e and Supplementary Video 1, laboratory studies showed a total sniff duration of 85.7 ± 8.5 s in normal mice. SHH*5000* and SHH*8000* significantly increased sniff duration to 5.3 times (Supplementary Video 2) and 4.3 times (Supplementary Video 3) as much as that of normal mice (*P* = 0.0144 and *P* = 0.0462), respectively.

In Fig. 1f and Supplementary Video 1, laboratory studies showed a total rear supported duration of 18.7 ± 1.8 s and number of 12.0 ± 2.3 in normal mice. Unexpectedly, SHH*5000* and SHH*8000* significantly increased rear supported duration to 6.0 times (Supplementary Video 2) and 5.2 times (Supplementary Video 3) as much as that of normal mice, respectively. SHH*5000* significantly increased rear supported number to 9.3 times as much as that of normal mice. SHH*8000* could not significantly increase rear supported number.

Finally, as shown in Fig. 1g, laboratory studies showed a total rear unsupported duration of 35.7 ± 3.5 s and number of 47.0 ± 3.5 in normal mice (Supplementary Video 1). SHH*5000* and SHH*8000* significantly increased the duration to 5.2 and 3.6 times, and increased the number to 3.2 and 2.7 times, respectively. There was no significant difference between the increase by SHH*5000* and SHH*8000.*

In conclusion, SHH improved mice locomotor under SHH condition. Effects of mild SHH is higher than severe SHH.

### Results of rotarod tests and elevated plus maze (EPM) tests

**Figure 2:**
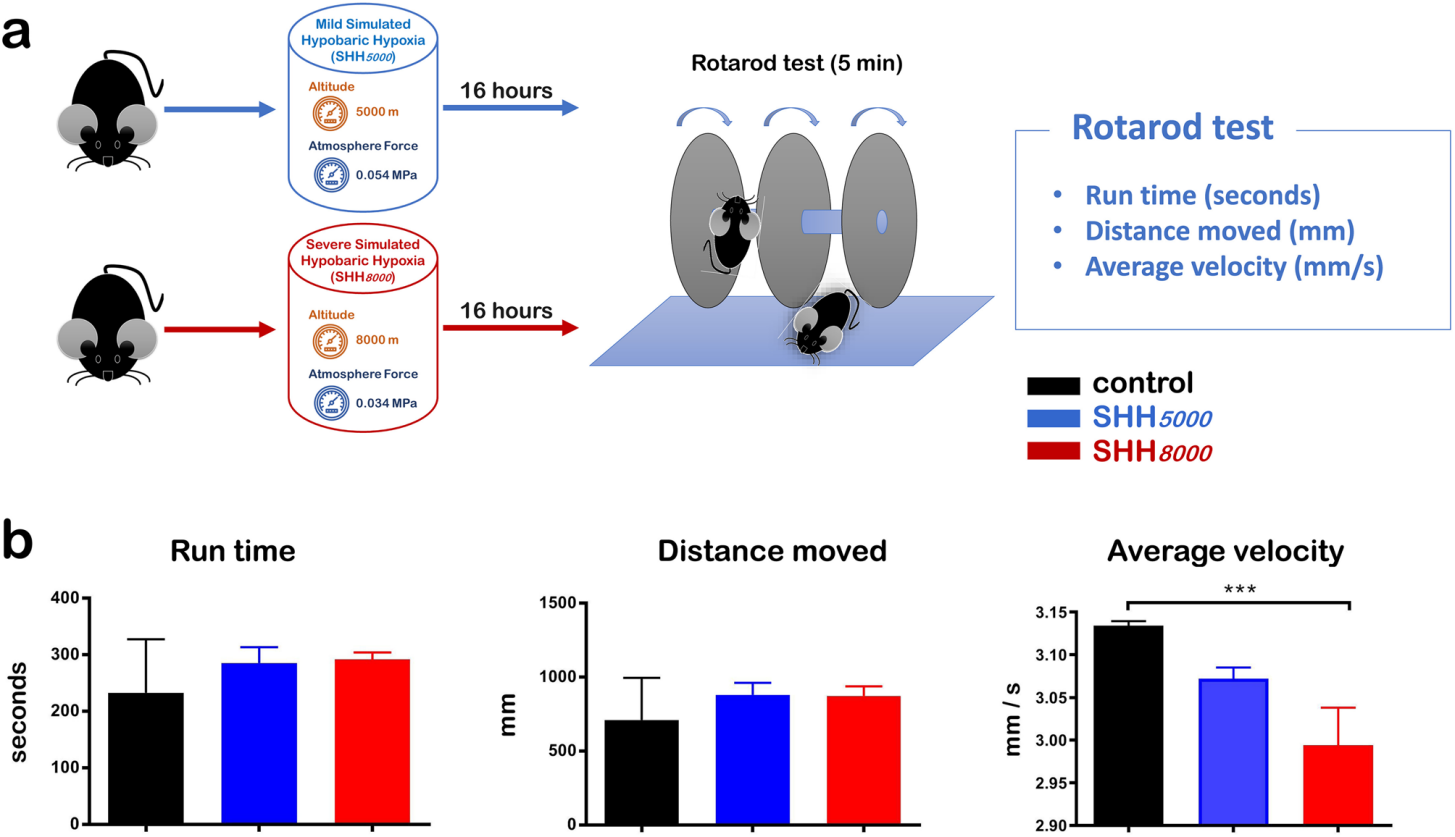
Mice presented an improved balance ability under SHH conditions. (**a**) Scheme of the rotarod test after 16 h of SHH*5000* or SHH*8000 *treatment aiming to examine the balance ability of mice. (**b**) Run time, distance and average speed after SHH*5000* and SHH*8000* treatment. Each value is expressed as the mean ± standard error of the mean. Multiple comparisons between groups were carried out with Tukey’s post hoc test. *P value < 0.05; **P value < 0.01; ***P value < 0.001, n = 5 per group.

In order to observe the effects of the SHH on the balance ability of mice, rotarod test was performed (Fig. 2a). As shown in Fig. 2b, both SHH*5000* and SHH*8000* could not change the two items significantly. Moreover, in the 5 min duration time of rotarod tests, the average velocity in normal mice decreased to be 95.0% in mice of SHH*8000* group (*P* = 0.0022); although SHH*5000* could not significantly change the average velocity.

**Figure 3:**
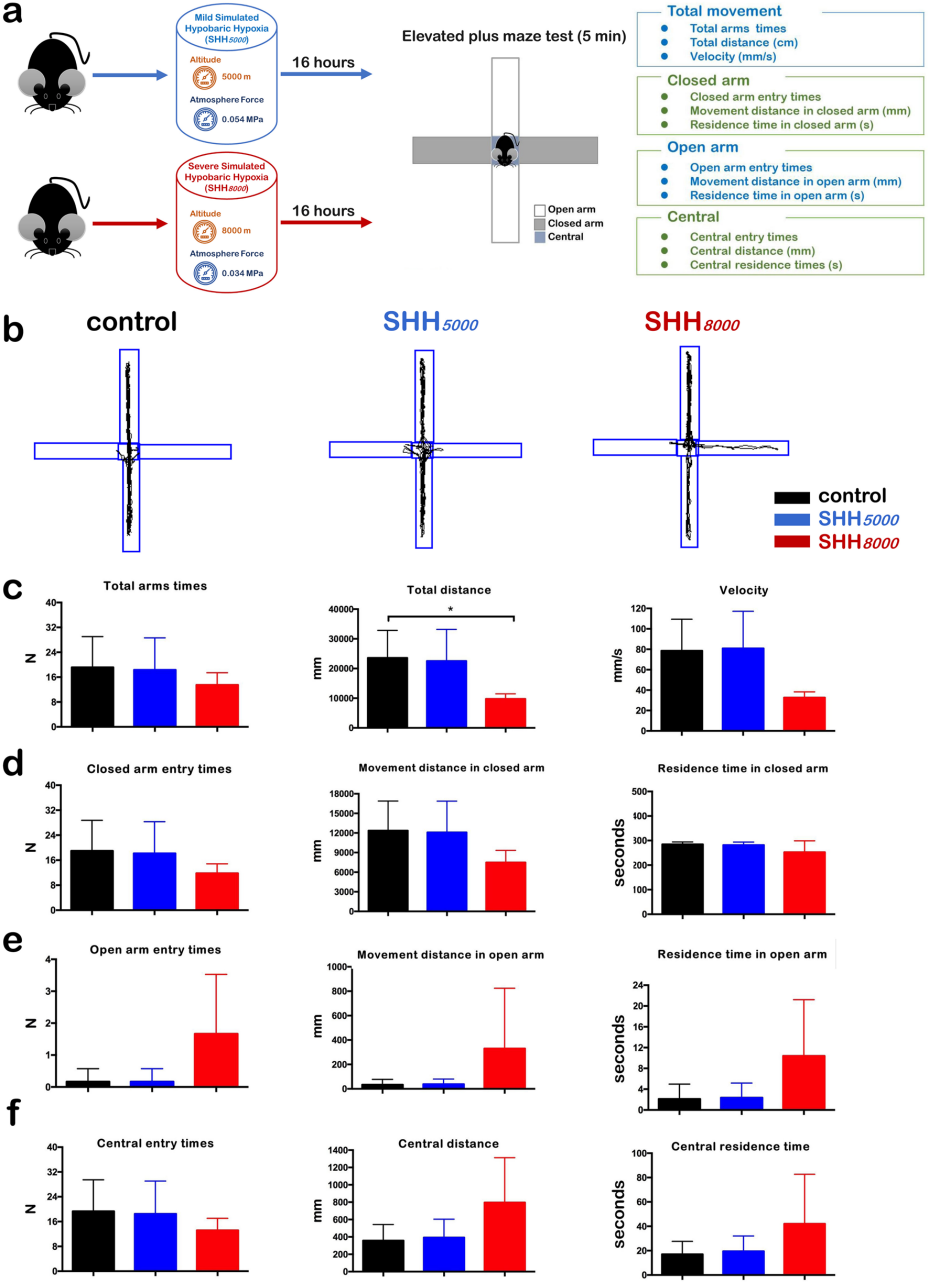
Mice presented improved exploratory behaviors under SHH conditions. (**a**) Scheme of the elevated plus maze test after 16 h of SHH*5000* or SHH*8000* treatment aiming to examine the exploratory behaviors of mice. (**b**) Trajectory pattern diagram after SHH*5000* or SHH*8000* treatment. (**c**) Total arms times, total distance and speed (from left to right). (**d**) Closed arm entry times, movement distance in the closed arm and residence time in the closed arm (from left to right). (**e**) Open arm entry times, movement distance in the open arm and residence time in the open arm (from left to right). (**f**) Central entry times, central distance and central residence time (from left to right). Each value is expressed as the mean ± standard error of the mean. Multiple comparisons between groups were carried out with Tukey’s post hoc test. *P value < 0.05; **P value < 0.01; ***P value < 0.001, n = 5 per group.

At the same time, the exploratory behaviors of SHH mice were assessed by elevated plus maze (EPM) test (Fig. 3a,b). Compared to control group, all eleven items of mice after SHH*5000* treatment or SHH*8000* treatment did not show significant difference, including total arms time and velocity in Fig. 3c, three items in closed arm (Fig. 3d), three items in open arm (Fig. 3e), and three items in central area (Fig. 3f). Only the total distance (mm) of normal mice decreased to be 41.5% in mice of SHH*8000* group; although SHH*5000* could not significantly change it (Fig. 3c).

### In cerebellum, 6 of all 8 detected mitochondria-related genes did not change after a single SHH

**Figure 4:**
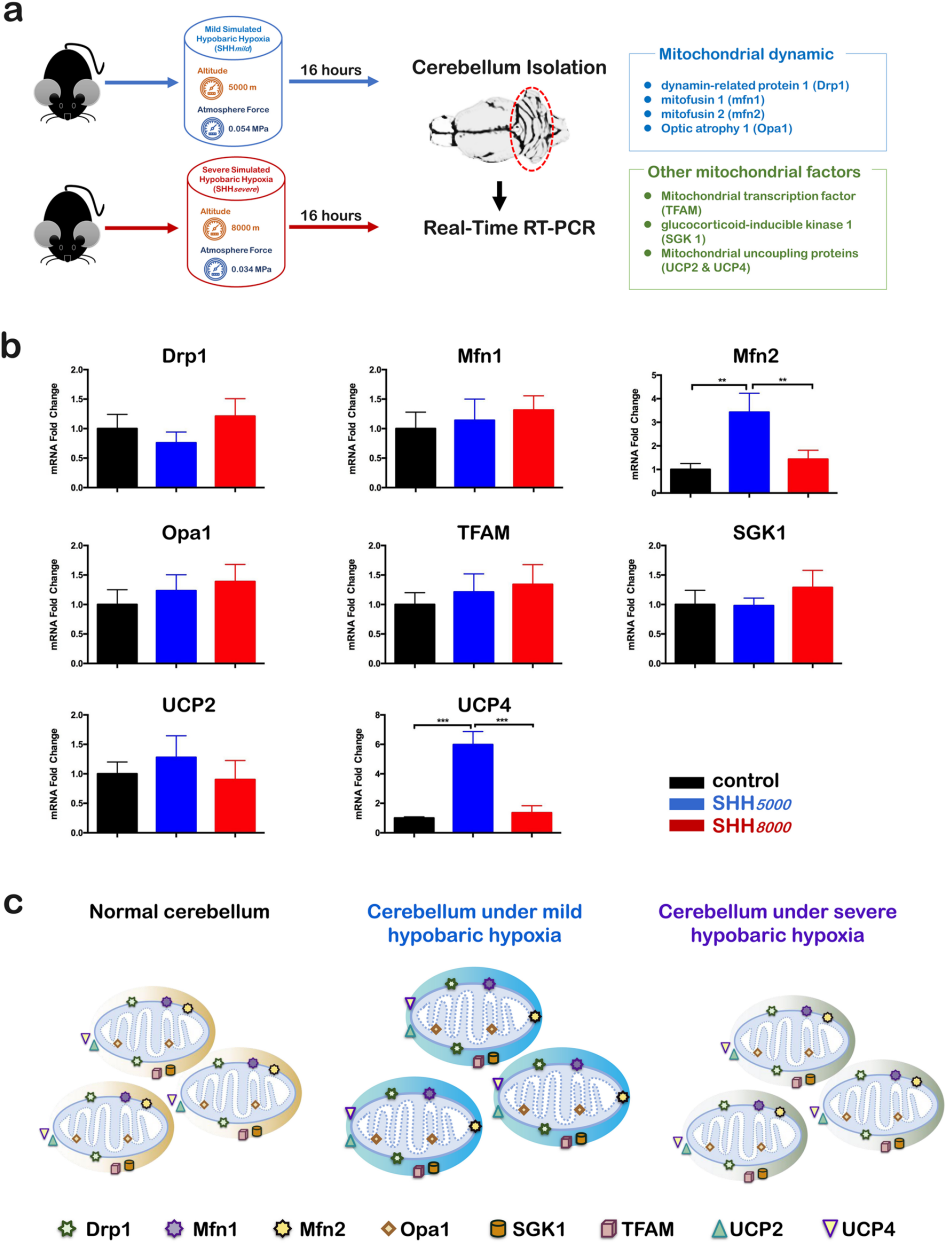
In the cerebellum of mice with simulated hypobaric hypoxia treatment, only the levels of Mfn2 and UCP4 were statistically increased by SHH*5000*, whereas all other genes were almost the same as those in the normal control group. (**a**) Normal mice were sacrificed at SHH*5000* or SHH*8000* for 16 h. The mice were sacrificed and the cerebellum was collected, immediately stored in liquid nitrogen. Real-time quantitative PCR was then performed to detect mitochondrial-related molecules (Drp1, Mfn1, Mfn2, Opa1, TFAM, SGK1, UCP2, UCP4) mRNA expression levels differences. (**b**) Simulated hypobaric hypoxia induced mitochondrial dynamic altering in cerebellum of mice. qRT-PCR analyses of Drp1, Mfn1, Mfn2, Opa1, TFAM, SGK1, UCP2, UCP4 in cerebellum of mice. In the cerebellum of mice with SHH treatment, only the levels of Mfn2 and UCP4 were statistically increased by SHH*5000*, whereas all other genes, including DRP1, other mfn1 and Opa1, TFAM, SGK1 and UCP2 were almost the same as those in the normal control group. Data are presented as mean SD. *P < 0.05, **P < 0.01, ***P < 0.001, compared with the control group. n = 5 per group. (**c**) Schematic depiction of the mitochondrial dynamics within the cerebellum of mice in different conditions including normal control, exposed at SHH*5000* and SHH*8000*, respectively. It can be found the mRNAs levels of six mitochondria-related genes including Drp1, Mfn1, Opa1, SGK1, TFAM and UCP2 remained stable except that the mRNAs levels of Mfn2 and UCP4 increased significantly only at SHH*5000*. Drp1, dynamin-related protein 1, mitochondrial fission factor on the mitochondrial outer membrane; Mfn1, mitochondrial fusion protein 1, mitochondrial fusion factor on the mitochondrial outer membrane; Mfn2, mitochondrial fusion protein 2, mitochondrial fusion factor on the mitochondrial outer membrane; Opa1, Optic atrophy 1, mitochondrial fusion factor on the mitochondrial inner membrane; SGK1, serum and glucocorticoid-regulated protein kinase 1, mitophagy-related factor; TFAM, mitochondrial transcription factor; UCP2, uncoupling protein 2, mitochondrial stress factor; UCP4, uncoupling protein 4, mitochondrial stress factor; SHH*5000*, simulated hypobaric hypoxia at altitude 5000 m; SHH*8000*, simulated hypobaric hypoxia at altitude 8000 m.

We further investigated whether hypoxia-treated mice showed any changes in multiple mitochondrial genes, including Drp1, Mfn1, Mfn2, Opa1, TFAM, SGK1, UCP2 and UCP4 in cerebellum by qRT-PCRs (Fig. 4a). It was shown that SHH*5000* increased significantly the levels of two genes, Mfn2 (*P* = 0.0124) and UCP4 (*P* < 0.0001), but did not change the mRNA levels of Drp1, Mfn1, Opa1, TFAM, SGK1, and UCP2 (Fig. 4b). Therefore, only the levels of Mfn2 and UCP4 were significantly increased in cerebellum under SHH*5000*, whereas the levels of other genes in either SHH*5000* or SHH*8000* were detected no changes (Fig. 4c).

### In hippocampus, all 8 detected mitochondria-related genes did not change after a single SHH

**Figure 5:**
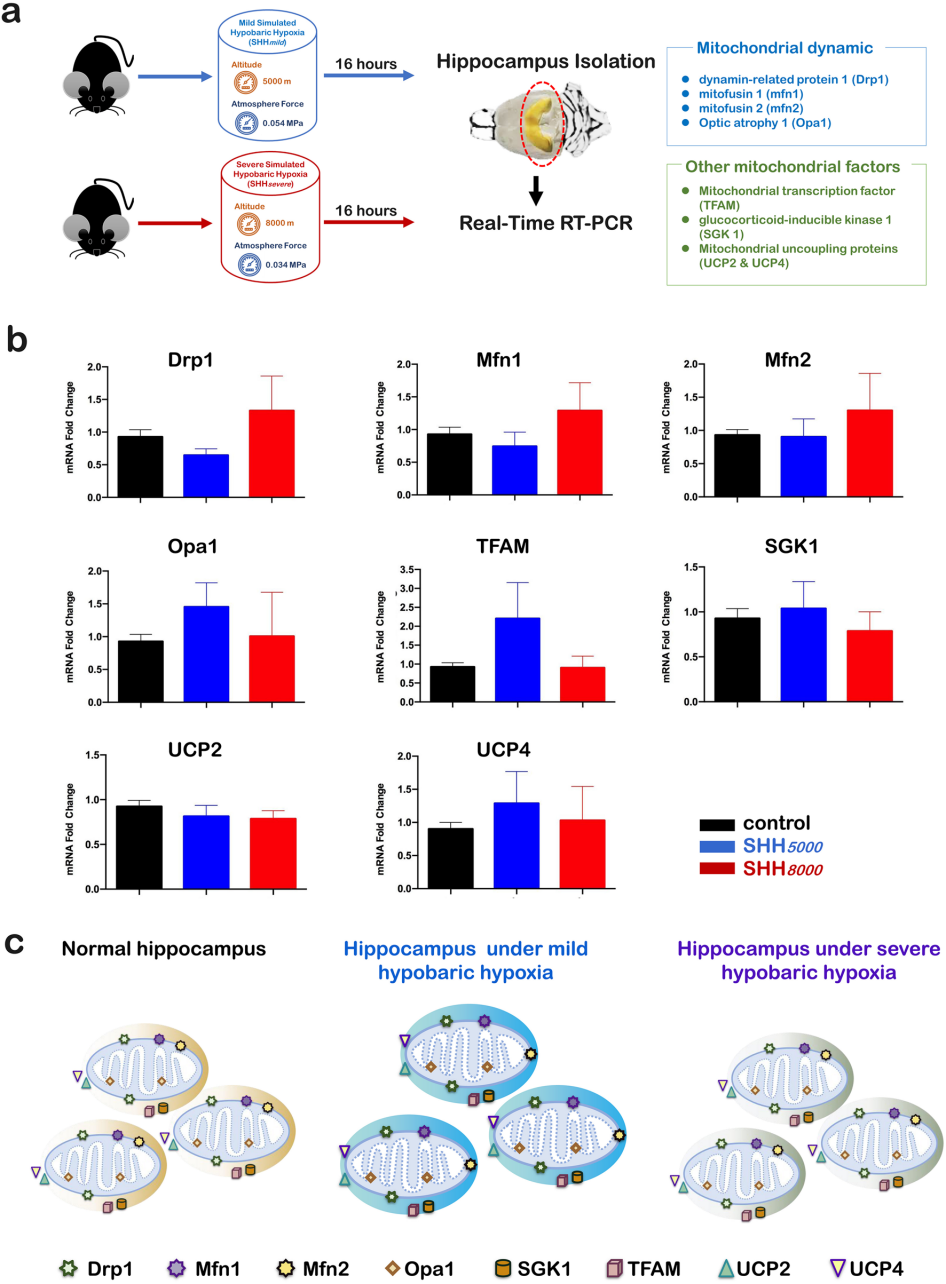
In the hippocampus, no changes in any gene mRNA levels were detected after SHH*5000* and SHH*8000* treatment. (**a**) Normal mice were sacrificed after SHH*5000* or SHH*8000* treatment for 16 h. The mice were sacrificed, and each hippocampus was collected and immediately stored in liquid nitrogen. Real-time quantitative PCR was then performed to detect differences in the mRNA expression levels of mitochondrial-related genes (Drp1, Mfn1, Mfn2, Opa1, TFAM, SGK1, UCP2, UCP4). (**b**) Simulated hypobaric hypoxia-induced mitochondrial dynamic alterations in the hippocampus of mice. qRT-PCR analyses of Drp1, Mfn1, Mfn2, Opa1, TFAM, SGK1, UCP2, and UCP4 in the hippocampus of mice. In the hippocampus of mice after SHH treatment, no changes in the mRNA levels of any of the genes examined were detected. Data are presented as the mean ± SD. *P < 0.05, **P < 0.01, ***P < 0.001, compared with the control group. n = 5 per group. (**c**) Schematic depiction of the mitochondrial dynamics within the hippocampus of mice after different treatments, including the normal control and after to SHH*5000* and SHH*8000* treatment. Drp1, dynamin-related protein 1, a mitochondrial fission factor on the mitochondrial outer membrane; Mfn1, mitochondrial fusion protein 1, a mitochondrial fusion factor on the mitochondrial outer membrane; Mfn2, mitochondrial fusion protein 2, a mitochondrial fusion factor on the mitochondrial outer membrane; Opa1, Optic atrophy1, a mitochondrial fusion factor on the mitochondrial inner membrane; SGK1, serum and glucocorticoid-regulated protein kinase 1, a mitophagy-related factor; TFAM, mitochondrial transcription factor; UCP2, uncoupling protein 2, a mitochondrial stress factor; UCP4, uncoupling protein 4, a mitochondrial stress factor; SHH*5000*, simulated hypobaric hypoxia at an altitude of 5000 m; SHH*8000*, simulated hypobaric hypoxia at an altitude of 8000 m.

Also the changes of mRNAs in 8 mitochondria-related genes in hippocampus were detected by qRT-PCRs (Fig. 5a). As shown in Fig. 5b, it was shown that both SHH*5000* and SHH*8000* did not increase or decrease the levels of Drp1, Mfn1, Mfn2, Opa1, TFAM, SGK1, UCP2, and UCP4. There was also no significant difference between the increase by SHH*5000* and SHH*8000.* Therefore, all eight detected mitochondria-related genes within the hippocampus maintained stable under both SHH*5000* and SHH*8000* conditions (Fig. 5c).

### In gastrocnemius muscle, 7 of all 8 detected mitochondria-related genes did not change after a single SHH

**Figure 6:**
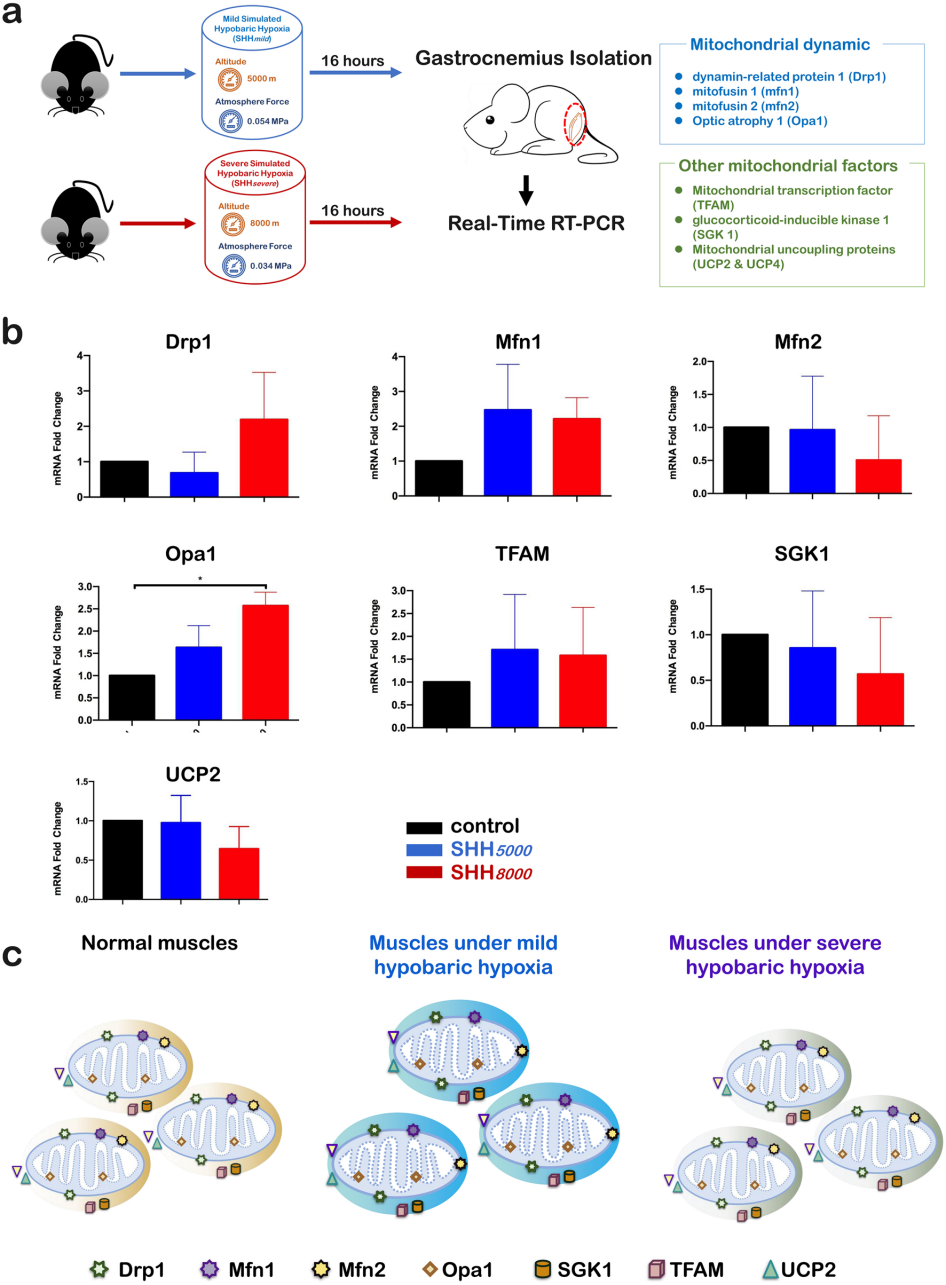
Only the levels of Opa1 significantly increased in the muscles after SHH*8000* treatment, whereas the levels of the other examined genes in the muscles showed no changes. (**a**) Normal mice were sacrificed after SHH*5000* or SHH*8000* treatment for 16 h. The muscles were collected and immediately stored in liquid nitrogen. Real-time quantitative PCR was then performed to detect differences in the mRNA expression levels of mitochondrial-related genes (Drp1, Mfn1, Mfn2, Opa1, TFAM, SGK1, UCP2, and UCP4). (**b**) Simulated hypobaric hypoxia-induced mitochondrial dynamic alterations in the muscles of mice. qRT-PCR analyses of Drp1, Mfn1, Mfn2, Opa1, TFAM, SGK1, UCP2, and UCP4 in the muscles of mice. In the muscles of mice with SHH treatment, only the level of Opa1 was significantly increased by SHH*8000* treatment, whereas the levels of all other genes examined in muscles and all genes examined in the hippocampus showed no changes compared to the control. Data are presented as the mean ± SD. *P < 0.05, **P < 0.01, ***P < 0.001, compared with the control group, n = 5 per group. (**c**) Schematic depiction of the mitochondrial dynamics within the cerebellum of mice in different conditions including normal control, exposed at SHH*5000* and SHH*8000*, respectively. It can be found the mRNAs levels of six mitochondria-related genes including Drp1, Mfn1, Opa1, SGK1, TFAM and UCP2 remained stable except that the mRNAs levels of Mfn2 and UCP4 increased significantly only at SHH*5000*. Drp1, dynamic-related protein 1, mitochondrial fission factor on the mitochondrial outer membrane; Mfn1, mitochondrial fusion protein 1, mitochondrial fusion factor on the mitochondrial outer membrane; Mfn2, mitochondrial fusion protein 2, mitochondrial fusion factor on the mitochondrial outer membrane; Opa1, Optic atrophy1, mitochondrial fusion factor on the mitochondrial inner membrane; SGK1, serum and glucocorticoid-regulated protein kinase 1, mitophagy-related factor; TFAM, mitochondrial transcription factor; UCP2, uncoupling protein 2, mitochondrial stress factor; UCP4, uncoupling protein 4, mitochondrial stress factor; SHH*5000*, simulated hypobaric hypoxia at altitude 5000 m; SHH*8000*, simulated hypobaric hypoxia at altitude 8000 m.

In addition, the eight mitochondrial genes in gastrocnemius muscle were detected by qRT-PCRs (Fig. 6a). It should be indicated that UCP4 was a brain-specific mitochondrial protein, which could not be detected in the skeletal muscles. The present qRT-PCR results have confirmed UCP4 mRNAs could not be detected at normal control and SHH conditions. And as shown in Fig. 6b, it was shown that both SHH*5000* and SHH*8000* did not increase or decrease the levels of Drp1, Mfn1, Mfn2, Opa1, TFAM, SGK1 and UCP2. Therefore, all eight detected mitochondria-related genes within the muscles maintained stable under both SHH*5000* and SHH*8000* conditions (Fig. 6c).

### Western blot results

**Figure 7:**
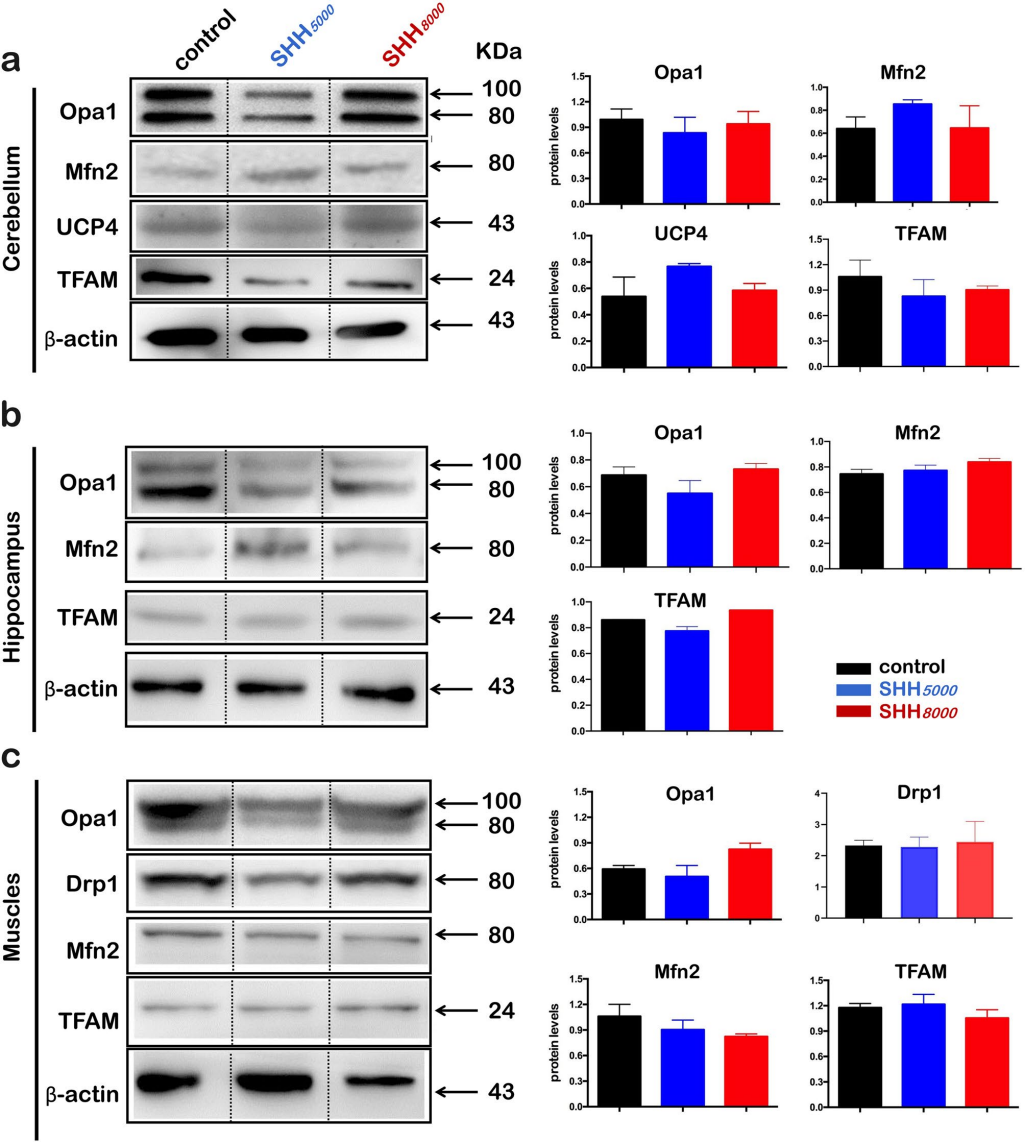
Protein expression levels showed a trend similar to those of the mRNA levels. (**a**) Normal mice were sacrificed after SHH*5000* or SHH*8000* treatment for 16 h. Each cerebellum was collected and immediately stored in liquid nitrogen. Western blotting was then performed to detect differences in mitochondrial-related protein expression levels (Mfn2, Opa1, TFAM, UCP4). (**b**) Western blotting was performed to detect differences in mitochondrial-related protein expression levels (Mfn2, Opa1, TFAM) in each mouse hippocampus after SHH*5000* or SHH*8000* treatment. (**c**) Western blotting was performed to detect differences in mitochondrial-related protein expression levels (Mfn2, Opa1, TFAM) in the muscles of mice after SHH*5000* or SHH*8000* treatment. Data are analyzed by Image J software, and are presented as the mean ± SD. *P < 0.05, **P < 0.01, ***P < 0.001, compared with the control group, n = 5 per group. Full-length blots/gels are presented in Supplementary Fig. 1a–c. Mfn2, mitochondrial fusion protein 2, a mitochondrial fusion factor on the mitochondrial outer membrane; Opa1, Optic atrophy1, a mitochondrial fusion factor on the mitochondrial inner membrane; TFAM, mitochondrial transcription factor; UCP4, uncoupling protein 4, a mitochondrial stress factor; SHH*5000*,simulated hypobaric hypoxia at an altitude of 5000 m; SHH*8000*, simulated hypobaric hypoxia at an altitude of 8000 m.

Because we have detected the significant increases of mRNAs of Mfn2 & UCP4 in cerebellum, as well as Opa1 in gastrocnemius muscle, we further performed Western blot to explore the protein expression changes of five mitochondria-related genes, including Drp1, Mfn2, Opa1, TFAM and UCP4, in the cerebellum, hippocampus and gastrocnemius muscle tissues (Fig. 7). Opa1 protein level in muscle tissues under SHH*8000* is higher than other group, but with no significant difference (Fig. 7c). All other four protein levels under both SHH*5000* and SHH*8000* did not change whenever in cerebellum (Fig. 7a), hippocampus (Fig. 7b) and gastrocnemius muscle tissues (Fig. 7c).

### Morphology of cerebellar mitochondria did not change after SHH treatment

**Figure 8:**
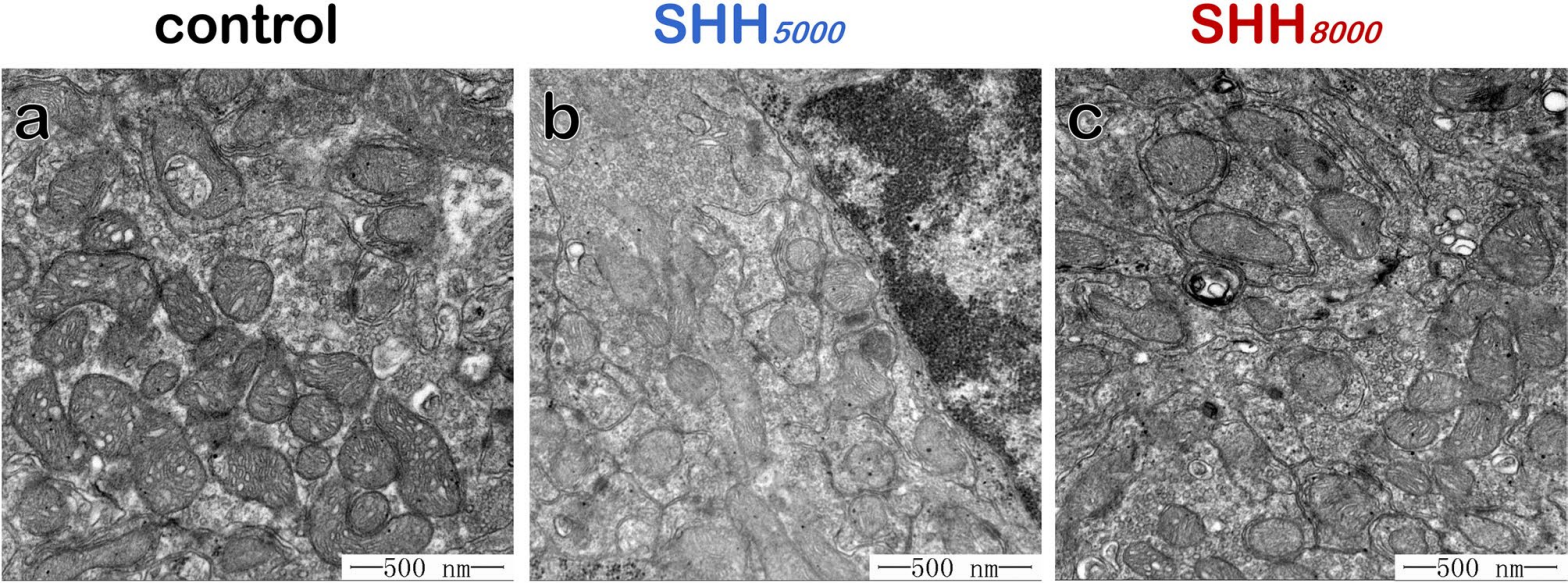
Representative transmission electron microscopy (TEM) images of cerebellar mitochondria. The circular, granular and tubular shaped mitochondria were frequently observed in the mice in normal control group (**a**), SHH*5000* group (**b**), and SHH*8000* group (**c**). Results showed that mitochondrial morphology in cerebellum did not significantly change after SHH treatment. Scale bars, 500 nm.

Since the Mfn2, UCP4 were found enhancing after SHH treatment, we further observed mitochondrial structure in cerebellum under TEM after SHH treatment. Results showed that mitochondrial morphology in cerebellum did not significantly change after SHH*5000* (Fig. 8b) or SHH*8000* (Fig. 8c) treatment, compared with that in normal control group (Fig. 8a).

## Discussion

The current study used a mouse decompression chamber to simulate mild hypobaric hypoxia at the high altitude of 5000 m or severe hypobaric hypoxia at 8000 m for 16 h (SHH*5000 &* SHH*8000*, respectively). Firstly, it has been shown that a single mild or severe SHH could improve healthy mice performance by using a combination of modified Noldus video tracking system, rotarod test and elevated plus maze (EPM) test. Secondly, the results by using qRT-PCR and Western blot have shown that 5 important mitochondria-related genes of Drp1, Mfn1, TFAM, SGK1 and UCP2 haven’t significant changes in cerebellum, hippocampus or gastrocnemius muscle after SHH. Thirdly, cerebellar mitochondria detected by transmission electron microscopy (TEM) haven’t morphological changes after SHH treatment. The present study has indicated the benefit of a single SHH in healthy mice performance, which would due to the stabilized mitochondria against a mild stress state.

The present beneficial effect of SHH on performance is seemingly counterintuitive. In fact, hypobaric hypoxia have long been used for training pilots, mountaineers, and athletes and even applied for the treatment and prevention of human diseases such as hypertension^14^, ischemic coronary artery diseases^20^, Parkinson’s disease^21^, and acute myeloid leukemia^22^. Kiers D et al. have demonstrated that short-term hypoxia (3.5 h) dampens the systemic pro-inflammatory cytokine response through enhancing purinergic signaling in mice and men^23^. Serebrovska et al. have revealed that intermittent hypoxia-hyperoxia training improves cognitive function and decreases circulating biomarkers of Alzheimer’s disease. Similarly, hypoxic exposure can enhance both physical and mental capacities, resulting in increased aerobic performance capacity^24^*.* Evidence has demonstrated that mild hypoxic preconditioning and hypoxia-mimetic agents are beneficial to cerebral stroke and some neurodegenerative disorders^25^. Recently, it has been shown that hypoxia promotes neurogenesis in vitro^26^. All these results have indicated a role of a single hypoxia exposure for the potential therapeutic effects, at least on mice.

It is notable that in the rotarod tests, mice with SHH*8000* treatment had decreased average velocity; also in the EPM tests, mice with SHH*8000* treatment had decreased total distance. But we should consider the duration of both rotarod and EPM tests is only within 5 min, which is much shorter than the duration of 60 min in the modified Noldus video tracking system. Our Noldus recordings had evidenced that the distance moved, the sniff number and the rear-supported number indeed decreased at the early 8 min (indicated by the hollow arrow in Fig. 1). However, after a short recovery from the hypoxia, these three items began to increase and exceeded the normal level after then on. Some research has reported that short-term athletic training at an altitude of 1860 m can significantly improve 150 m running speed times^27^. And a single short-term traditional “live high-train high” or contemporary “live high-train low” training will lead to a 3% increase in O_2_ transportation and a 1.1–1.2% improve in sea level 3000 m run time^8^. Based on the data, it is tempting to speculate SHH indeed has beneficial effect on performance outcome.

Mitochondria-related genes are an important basis for the homeostasis of the central nervous system and physiological functions. Once the body is stimulated by extreme environments, mitochondrial UCPs immediately function to ensure normal mitochondrial function and maintain the body's normal living environment. Hypoxia can be regarded as an extreme environment that is accompanied by physiological metabolic challenges in normal tissues. Exercise or training increases expression levels of UCP2^28^ and UCP3^29^. Previous studies have shown that the UCP4 expression level in the cerebellum may potentially function as a protective factor to enhance neuronal survival^30^. The present qRT-PCR results showed that, after SHH*5000* treatment, only the levels of Mfn2 and UCP4 within the cerebellum increased; in both hippocampus and gastrocnemius muscle, all 8 detected mitochondria-related genes did not change. UCP4 is a brain-specific isoform and its function in the neuron have not been established yet. Previous studies reported that UCP4 exhibits a competence of decrease mitochondrial membrane potential in cultured cells in vitro^19^. Xu et al. have found that UCP4 could lead to enhanced proton leak in cerebral mitochondrial membrane and stabilize mitochondrial Ca^2+^ homeostasis^31^. Our ultrastructural analysis further indicates cerebellar mitochondria show normal characteristics and do not display vacuolization of the cristae or distorted morphology. So we propose that cerebellum is sensitive to the physiological metabolic challenges and the elevated UCP4 upon hypoxia might contribute to its neuroprotective actions.

Mitochondrial dynamics has been recently linked to energy demand and supply balance^32^. At the molecular level, mitochondrial fusion depends on three fusion proteins: Mfn1 and Mfn2 at outer mitochondrial membranes and Opa1 at inner mitochondrial membranes^32^. Mfn2 is more abundant in the brain and skeletal muscle. Previous studies have shown that Mfn2 agonists could reverse mitochondrial defects in preclinical models of neurodegenerative disease of Charcot Marie Tooth disease type 2A (CMT2A) by mechanistically ameliorating mitochondrial trafficking^33^. The present results of the table Mfn2 mRNA levels within hippocampus and gastrocnemius tissues after SHH*5000* and SHH*8000,* as well as the cerebellum after SHH*8000*, may imply that the mitochondrial fusion or trafficking in these areas maintains in balanced state after SHH stress.

Opa1 can promote the fusion of mitochondria^32^ and the defect of the Opa1 gene leads to the malformation of the mitochondrial cristae morphology, which has a serious influence on the proliferation of the cells^34^. Patten et al. have revealed that Opa1-dependent modulation of cristae structure is necessary for cellular adaptation to energy substrate availability and is required for cell survival^34^. The present results of the stable Opa1 mRNA level, as well as unchanged mitochondrial morphology and cristae structures, within the cerebellum after both SHH*5000* and SHH*8000,* may imply the benefit of a single SHH in healthy mice performance is due to mitochondrial homeostasis. Rizo-Roca et al. have reported that the soleus muscles of the rats with 7 days exposure to intermittent hypobaric hypoxia at 4000 m have increased Opa1 protein expressions and activities^35^. We have also observed increased Opa1 mRNA level within the gastrocnemius skeletal tissues after SHH*8000.* These results would suggest a protective role of Opa1 against oxidative stress.

However, the present beneficial effect of SHH is challenged by two papers. Firstly, Chitra et al. have reported that the rats exposed to acute and severe hypobaric hypoxia (9142 m, 6 h) have had pulmonary oedema and the decreased Mfn2 expressions in lung tissue^36^. The controversy might be due to two points: altitudes and animal types. The hyobaric condition used by Chitra et al. has simulated much higher altitude (more than 9000 m) than that in the present study (SHH*5000* and SHH*8000* equal to 5000 and 8000 m, respectively). In addition, the study of Chitra et al. has imposed on rats, the present study on mice. Secondly, Zou et al. have reported that the rats subjected to SHH condition (5000 m) have had the decreased physical performance, as well as the decreased Mfn1 & Mfn2 mRNAs and increased drp1 mRNA in gastrocnemius tissues^37^. The controversy might be due to two points: SHH duration and animal types. The SHH used by Zou et al. lasted for 24 h, which was longer than the duration of 16 h in the present study. Also, the study of Zou et al. has imposed on rats. More importantly, we have noticed that the poor performance provided by Zou et al. is based on the results of rotarod tests. We have explained above that the duration of rotarod test is only within 5 min, which is much shorter than the duration of 60 min in the present modified Noldus video tracking system. We think the better physical performance under SHH condition in the present study is convincing.

In summary, the present findings open the door for further studies in hypobaric hypoxia effect in the field of the central nervous system and muscles. The benefit of a single SHH in healthy mice performance would due to the stabilized mitochondria against a mild stress state. Considering of the fact that being in the simulated hypobaric chambers has been already used in humans to improve exercise performance, it is deserved to detect the exact effects of SHH on mitochondrial dynamics, biogenesis, and activity of electron transporter chain.

## Materials and methods

### Mice

Adult male C57BL/6J mice with an average body weight of 20 ± 25 g were obtained from the Experimental Animal Center of the Air Force Medical University, Xi'an, Shaanxi, China. Mice were maintained on a 12:12 h light/dark cycle at 25 ± 1 °C with free access to standard laboratory feed and water. All animal protocols were approved by the Ethical Committee of the Air Force Medical University (permission number IACUC-20190107) and followed our institutional guidelines for the use of laboratory animals.

### Experimental design

To explore the influence of mild to severe SHH, mice were randomly divided into three groups (n = 35 pergroup): control normoxia group (control), mild SHH group (SHH*5000*), and severe SHH group (SHH*8000*). All mice but those in the control group were subjected to SHH for 16 h. Following treatment, 20 mice (n = 20) in each group were used for behavioral tests, and 15 mice (n = 15) in each group were sacrificed for tissue collections. In behavioral tests, 10 mice were video tracked to assess both their locomotor and exploratory behaviors (as shown in Fig. 1a), 5 mice measured by Rotarod test to assess their balance abilities (as shown in Fig. 2a), and 5 mice measured by Elevated plus maze test to assess their exploratory abilities (as shown in Fig. 3a). 20 mice were not sacrificed but be used for other relevant research by our research group other members.

We chose the humane endpoint after SHH exposure in mice, 15 mice in each group were humanely sacrificed with sodium pentobarbital (50 mg/kg body weight) by intraperitoneal injection and cervical dislocation. Then 10 mice were used for Quantitative Real Time—PCR (qRT-PCR) (as shown in Figs. 4a, 5a, 6a) and Western blot to analyze changes in mRNA and protein levels of mitochondria-related genes within the cerebellum, the hippocampus and the gastrocnemius muscle. At the same time, 5 mice were perfused for transmission electron microscopy (TEM) analysis.

### Simulated hypobaric hypoxia (SHH) treatment

To simulate a hypobaric hypoxia environment, a 12 mm thick stainless steel cabin (300 × 400 × 500 cm) was used^38^. An enclosed cabin, an exhaust valve and an intake valve leading into the cabin were installed and then connected to a vacuum pump. The hypobaric hypoxia cabin was designed by Professor Jin Ma, and the instrument was provided by the Department of Aerospace Medicine, Air Force Medical University, Xi’an, China. After normal mice were placed into the SHH chamber, the vacuum pump was turned on, and the gas in the cabin was continuously drawn out, resulting in low air pressure in the cabin. Environmental hypoxia was generated by adjusting the relative concentrations of nitrogen and oxygen in the input gas mixture. This created environmental oxygen tensions similar to those found in the high mountain communities of Nepaland Peru^39^*.* Continuous gas flow and CO_2_ absorption by Ca(OH)_2_ within the hypoxic chamber maintained CO_2_ levels below 0.4% with continuous monitoring. According to the method described by Paul H Holloway et al.^40^, the cabin pressure was adjusted to drop within 20 min to a vacuum of minus 11.01 kPa in the SHH*5000* group, which was equivalent to an altitude of 5000 m atmosphere force; and the cabin pressure was adjusted to drop within 20 min to a vacuum of minus 7.51 kPa in the SHH*8000* group, which was equivalent to an altitude of 8000 m atmosphere force. A control ambient environment for breathing 21% O_2_ was created with an identical chamber setup. During the experiment, the temperature was maintained at 20 ± 3 °C, and the humidity was 65 ± 5%.

### Modified Noldus video tracking and tests

To observe the behavioral changes of the mice after SHH exposure, locomotor functionswere assessed using a set of modified Noldus PhenoTyper (Model 3000) chambers (Leeburg, VA) with shock floors as previously described^41^ (as shown in Fig. 1A)***.***The PhenoTyper Model 3000 chamber has a 30 cm × 30 cm floor and is 40 cm in height. The PhenoTyper chamber is equipped with a top unit including a matrix of infrared LED lights and an infrared CCD camera with a high-pass filter blocking visible light. The floor of the cages was modified to include a stainless steel grid (interbar separation: 0.9 cm) connected to an electric shock generator (Shock Scrambler ENV-414S; Med Associates, St. Alvans; VT). Automated tracking and shock delivery control were performed using EthoVision 3.1 software (Noldus, Wageningen, the Netherlands)^42^. Individual mice were introduced into each chamber andacclimated to the apparatus for 60 min. Then, all mice were removed and left undisturbed inside their home cages. Subsequently, the mice were placed individually in the same cages they had experienced and were tested for 60 min. Behavioral data were collected, and then digital data were analyzed. Twelve items of observed behavioral elements were grouped into two categories. The first category is mainly related to locomotor activity and includes six items: distance moved (cm), average velocity (cm/s), walk duration (s), walk number (n), rest duration (s) and rest number (n). The walk state indicates that the mouse moves to another place with the hind legs moving as well. The rest state indicates resting without any movement, either sitting or lying down, including sleeping. The duration (s) and the number of walks or resting states were analyzed. The second category is mainly related to exploratory behavior, including duration (s) and number (n) of rear supported, rear unsupported or sniff states. The rear supported state indicates exploring while standing in an upright posture while leaning with the front paws against the cage wall or another object. The rear unsupported state indicates exploring while standing in an upright posture. The sniff state indicates slight movements of the head in order to gather information about the environment, possibly with slight, discontinuous displacement. The supplementary videos illustrate the performance of the behavioral tests.

### Rotarod test

To assess the influence of SHH on the balance ability of mice, the rotarod test was performed (as shown in Fig. 2a). The rotarod test system from Shanghai Jiliang Software Technology Co., Ltd (Shanghai, China) was used. Before the test, mice were trained for three successive days at a constant speed (8 rpm on the first day, 11 rpm on the second day and 15 rpm on the third day). For the test, the mice were placed on the rotarod apparatus, with rotation starting at 8 rpm and progressing to a maximum of 20 rpm. The maximum speed allowed was 300 s per trial, and the falling latency of the mice was recorded. Three items were assessed including run time (s), distance moved (mm) and average velocity (mm/s). Each mouse was measured three times.

### Elevated plus maze test

To assess the influence of SHH on the exploratory behavior of mice, the elevated plus maze test (Med Associates, St. Albans, Vermont) was performed (as shown in Fig. 3a). Animals were placed in the center square (10 × 10 cm) with their head towards a closed arm (40 × 10 cm), recorded for 5 min and analyzed with the motion tracking system. The total traveling distance and the central distance were recorded using the motion tracking system and analyzed by JLBehv-EPMG software (Shanghai Jiliang Software Technology Co., Ltd). Scoring was performed blinded to treatment type. Twelve items were assessed, including total arms times, total distance (cm), velocity (mm/s), closed arm entry times, movement distance in closed arm (mm), resistance time in closed arm (s), open arm entry times, movement distance in open arm (mm), resistance time in open arm (s), central entry times, central distance (mm), central resistance time (s). Each mouse was measured three times.

### Quantitative real time-PCR (qRT-PCR)

To assess the influence of SHH on 8 mitochondria-related genes, quantitative Real Time-PCR (qRT-PCR) was performed. The tissues of cerebellum (as shown in Fig. 4a), hippocampus (as shown in Fig. 5a) and gastrocnemius muscle (as shown in Fig. 6a) of mice in three groups were collected, homogenized and processed to measure total RNA using Trizol reagent (Invitrogen, CA) in accordance with the manufacturer's instructions. The corresponding cDNA was synthesized using the extracted RNA as a template and detected by qRT-PCR. The detected eight genes include4 mitochondrial dynamic-related genes of Drp1, Mfn1, Mfn2 and Opa1, as well as 4 other mitochondrial factors of TFAM, SGK1, UCP2 and UCP4. The primer sequences are shown in Table 1. The internal reference is β-actin (Biotech, Shanghai). The reaction conditions: set the program as a two-step real-time quantification, pre-denaturation 95 °C, 15 s, and then each step denatures 95 °C, 5 s, annealing, extension 60 °C for 30 s for 40 cycles. After amplification, a dissolution curve was performed to check the homogeneity of the product. The relative content was calculated using the 2^−△△Ct^ method for statistical analysis.

**Table 1:** Sequences of the primers employed for amplification of mRNAs encoding Drp1, Mfn1, Mfn2, Opa1, TFAM, SGK1, UCP2 and UCP4 by qRT-PCR.

mRNA	Sequences (5′–3′)		Length	Annealing temperature (°C)
Drp1	Forward: Reverse:	5′-AACAGGCAACTGGAGAGGAA-3′ 5′-GCAACTGGAACTGGCACAT-3′	144	58
Mfn1	Forward: Reverse:	5′-GGTCTGCTTTCCTGCTCTCT-3′ 5′-CTTTCTGCTCCCATTTCACC-3′	117	60
Mfn2	Forward: Reverse:	5′-CCTGGGATCGATGTTACCAC-3′ 5′-AACTGCTTCTCCGTCTGCAT-3′	119	62
Opa1	Forward: Reverse:	5′-GCCTTCCTCTTCGTCTCTCC-3′ 5′-CTCACTTGCTTCCACACCAA-3′	111	60
TFAM	Forward: Reverse:	5′-GCAGGCACTACAGCGATACA-3′ 5′-TACCTTTCCCATTCCCTTCC-3′	128	58
SGK1	Forward: Reverse:	5′-GGTGGACTGGTGGTGTCTTG-3′ 5′-GAAGGAATCCACAGGAGGTGC-3′	122	62
UCP2	Forward: Reverse:	5′-TGGGAGGTAGCAGGAAATCA-3′ 5′-GCGGTATCCAGAGGGAAAGT-3′	135	60
UCP4	Forward: Reverse:	5′-CTCAGAGCCAACCGAATAGC-3′ 5′-GGCTGACAGATGCAACAGAA-3′	142	58

### Western blotting

To assess the influence of SHH on mitochondria-related gene expressions, western blotting was performed. The tissues of cerebellum, hippocampus and gastrocnemius muscle of mice in three groups were collected into ice-cold homogenization buffer (50 mM Tris–HCl, pH 7.4, 150 mM NaCl, 1 mM EDTA, 0.5% Triton X, protease inhibitor cocktail) and homogenized before centrifugation (14,000*g*). Aliquots of the supernatant were incubated for 30 min at 37 °C in Laemmli SDS sample buffer (Invitrogen, Carlsbad, CA, USA). 20 μg of protein extracts were loaded on 10% acrylamide gel and blotted onto a methanol-activated PVDF membrane (Millipore, USA). The detected 5 gene proteins include 3 mitochondrial dynamic-related genes of Drp1, Mfn2 and Opa1, as well as 2 mitochondrial factors of TFAM and UCP4. Immunoblots were soaked in 5% nonfat milk 2 h at room temperature and subsequently probed with following primary antibodies overnight at 4 °C: rabbit-anti-Drp1 (1:250, Abcam, CA), rabbit-anti-Mfn2 (1:1000, Abcam, CA), rabbit-anti-Opa1 (1:1000, Abcam, CA), rabbit-anti-TFAM (1:1000, Abcam, CA), mouse-anti-UCP4 (1:200, Santa, CA), mouse-anti-β-actin (1:5000, Beijing, China). The immunoblots were then incubated with corresponding horseradish peroxidase (HRP)-conjugated secondary antibodies (1:5000, Beijing, China). The bands were detected with enhanced chemiluminescence (Beyotime, China) followed by exposure to luminometer (Bio-Rad, USA) and analyzed by ImageJ software. Target protein levels were normalized against β-actin levels and expressed as fold changes relative to those of the naive control group^43^.

### Transmission electron microscopy (TEM) analysis

To observe the mitochondrial morphological changes after SHH treatment, transmission electron microscopy (TEM) analysis within the cerebellum was performed. After anesthesia, mice were perfused with 0.01 M PBS and 4% formaldehyde with 0.1% glutaraldehyde in PBS successively. The cerebellums were removed and placed into 4% glutaraldehyde solution for tissue fixation. After fixation, each cerebellum was rinsed twice with 0.1 M PB. Then, 1% osmic acid (TED PELLA, Inc. No.18451) was used for staining for 2 h. Then, 50%, 70%, and 90% ethanol and 100% acetone were used successively for dehydration, followed by acetone: embedding agent (Embed812, DDSA, NMA, DMP-30) = 1: 1 for 2 h at room temperature. Finally, the cerebellar tissue was removed from the embedding agent and stored at room temperature overnight. On the next day, the tissue was placed into a special electron microscope plate at 60 °C for 48 h. An ultrathin microtome was used to make 70 nm thick slices. The slices were placed on a copper network and stained with lead nitrate and uranium acetate for 10 min each. Images were recorded using a transmission electron microscope (JEM1400, Olympus, Japan).

### Statistical analysis

Statistical analyses were performed with SPSS 15.0. Quantitative data are expressed as mean ± SD. The data are analyzed by one-way ANOVA (and nonparametric test). *P* < 0.05 was considered statistically significant.

## Supplementary Information

Supplementary Information 1.

Supplementary Video 1.

Supplementary Video 2.

Supplementary Video 3.

## Abbreviations

Drp1: Dynamic-related protein 1 (mitochondrial fission factor)
EPM: Elevated plus maze
HH: Hypobaric hypoxia
HIF: Hypoxia inducible factor
Mfn1: Mitochondrial fusion protein 1 (mitochondrial fusion factor)
Mfn2: Mitochondrial fusion protein 2 (mitochondrial fusion factor)
Opa1: Optic atrophy1 (mitochondrial fusion factor)
qRT-PCR: Quantitative real time-PCR
SGK1: Serum and glucocorticoid-regulated protein kinase 1 (mitophagy-related factor)
SHH: Simulated hypobaric hypoxia
SHH*5000*: Simulated hypobaric hypoxia 5000 m
SHH*8000*: Simulated hypobaric hypoxia 8000 m
TFAM: Mitochondrial transcription factor A
UCPs: Uncoupling proteins
UCP2: Uncoupling protein2 (mitochondrial stress factor)
UCP4: Uncoupling protein 4 (mitochondrial stress factor)
TEM: Transmission electron microscopy
