# Supplementary material for: Benefit of a single simulated hypobaric hypoxia in healthy mice performance and analysis of mitochondria-related gene changes
Source: Sci Rep. 2021 Feb 24;11:4494. doi: 10.1038/s41598-020-80425-8 (PMC7904831; doi:10.1038/s41598-020-80425-8)
Supplement: Supplementary file 1 — Supplementary Information 1. [file 41598_2020_80425_MOESM1_ESM.docx]

**Cover page**

**Title:**

Benefit of a single simulated hypobaric hypoxia in healthy mice performance and analysis of mitochondria-related gene changes

**Running Title:**

Benefit of hypoxia from stable mitochondrial dynamics

**Authors:**

Fei-Fei Wu1, **#** , Kun-Long Zhang2, **#**, Zheng-Mei Wang1,Yi Yang1

, Shao-Hua Li 3, Jia-Qi Wang1,Jin Ma3, Yan-Ling Yang 4, Hai-Feng Zhang1, Ya-Yun Wang1

**Author Affliations:**

*1. Specific Lab for Mitochondrial Plasticity Underlying Nervous System Diseases, National Demonstration Center for Experimental Preclinical Medicine Education, Air Force Medical University;*

*2. Department of Rehabilitation and Physical Therapy, Xi-Jing Hospital, Air Force Medical University, Xi’an 710032, China;*

*3. Department of Aerospace Physiology, Air Force Medical University, Xi’an 710032, China;*

*4. Department of Hepatobiliary Surgery, Xi-Jing Hospital, Air Force Medical University, Xi’an 710032, China.*

**# These authors contributed equally to this work.**

**# Fei-Fei Wu**

Master of Science in Biology & Teaching Assistant

Specific Lab for Mitochondrial Plasticity Underlying Nervous System Diseases, National Demonstration Center for Experimental Preclinical Medicine Education, Air Force Medical University, Xi’an 710032, China.

E-mail: wufeifei@fmmu.edu.cn

Office Tel: 0086-29-84774518

Mobile: 0086-13096908273

**# Kun-Long Zhang**

Bachelor of Medicine & Postgraduate Student, Department of Rehabilitation and Physical Therapy, Xi-Jing Hospital, Air Force Medical University, Xi’an 710032, China

E-mail: 983817468@qq.com

Office Tel: 0086-29-84775438

Mobile: 0086-15692544404

**Corresponding authors:**

**Ya-Yun Wang,**

Ph. D. & M.D.

Professor

Principle Investigator of Specific Lab for Mitochondrial Plasticity Underlying Nervous System Diseases,

Director of National Demonstration Center for Experimental Preclinical Medicine Education,

Air Force Medical University, Xi’an 710032, China.

E-mail: wangyy@fmmu.edu.cn; 462307338@qq.com

Office Tel: 0086-29-84712341

Mobile: 0086-13679168991****

**Hai-Feng Zhang,**

Ph. D. & M.D.

Assistant Professor

Specific Lab for Mitochondrial Plasticity Underlying Nervous System Diseases,

National Demonstration Center for Experimental Preclinical Medicine Education, Air Force Medical University, Xi’an 710032, China.

E-mail: hfzhang@fmmu.edu.cn

Office Tel: 0086-29-84779277;

Mobile: 0086-13669231260

**Yan-Ling Yang**

Ph. D. & M.D.

Professor

Department of Hepatobiliary Surgery,

Xi-Jing Hospital,

Air Force Medical University,

Xi’an 710032, China.

E-mail: yangyanl@fmmu.edu.cn

Office Tel: 0086-29-84775259

Mobile: 0086-13709246656

**Figure images captions and legends.**

**Supplementary fig 7. Protein expression levels showed by full-length blots/gels.**

**a,** Normal mice were sacrificed after SHH*5000* or SHH*8000* treatment for 16 h. Each cerebellum was collected and immediately stored in liquid nitrogen. Western blotting was then performed to detect differences in mitochondrial-related protein expression levels (Mfn2, Opa1, TFAM, UCP4).

**b,** Western blotting was performed to detect differences in mitochondrial-related protein expression levels (Mfn2, Opa1, TFAM) in each mouse hippocampus after SHH*5000* or SHH*8000* treatment.

**c,** Western blotting was performed to detect differences in mitochondrial-related protein expression levels (Mfn2, Opa1, TFAM) in the muscles of mice after SHH*5000* or SHH*8000* treatment.

Mfn2, mitochondrial fusion protein 2, a mitochondrial fusion factor on the mitochondrial outer membrane; Opa1, Optic atrophy1, a mitochondrial fusion factor on the mitochondrial inner membrane; TFAM, mitochondrial transcription factor; UCP4, uncoupling protein 4, a mitochondrial stress factor; SHH*5000*, simulated hypobaric hypoxia at an altitude of 5000 m; SHH*8000*, simulated hypobaric hypoxia at an altitude of 8000 m.


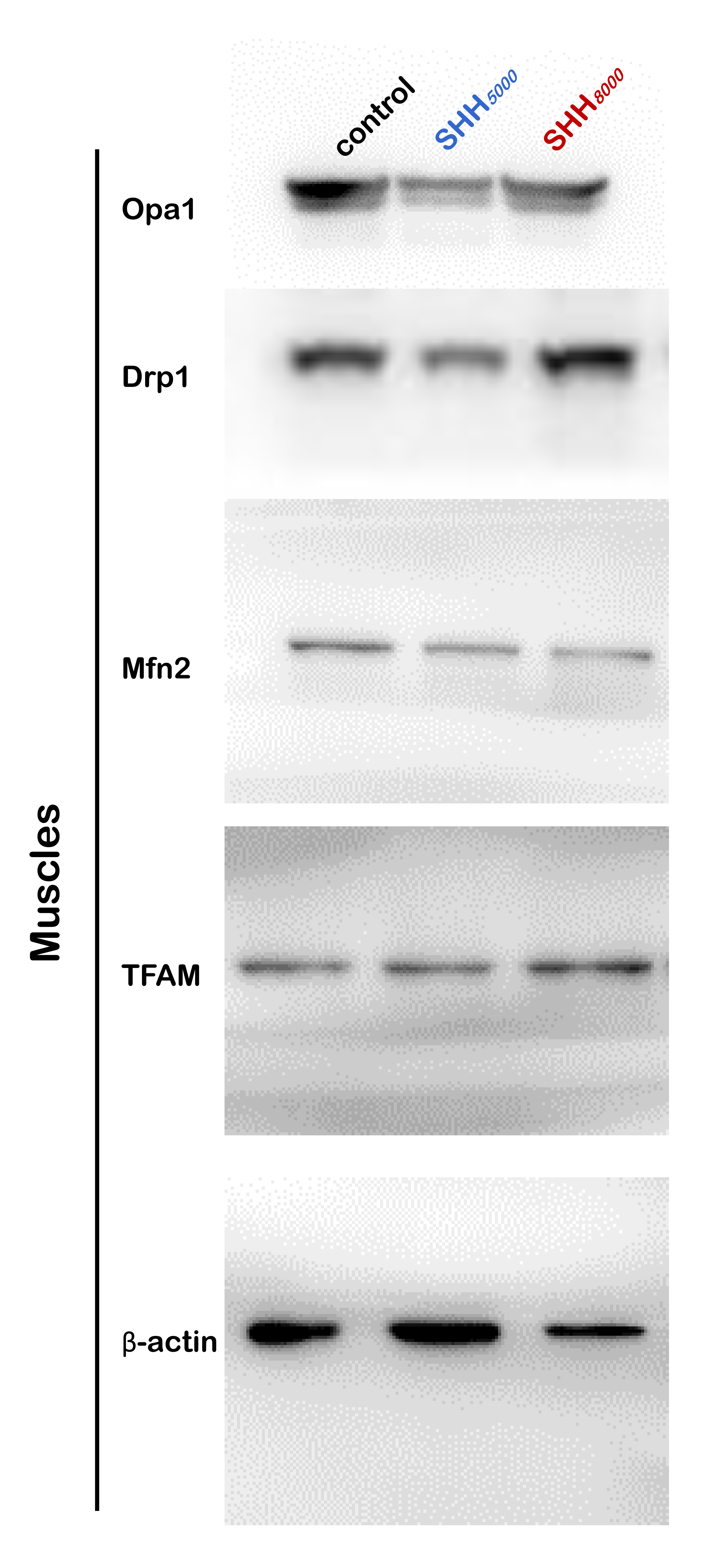

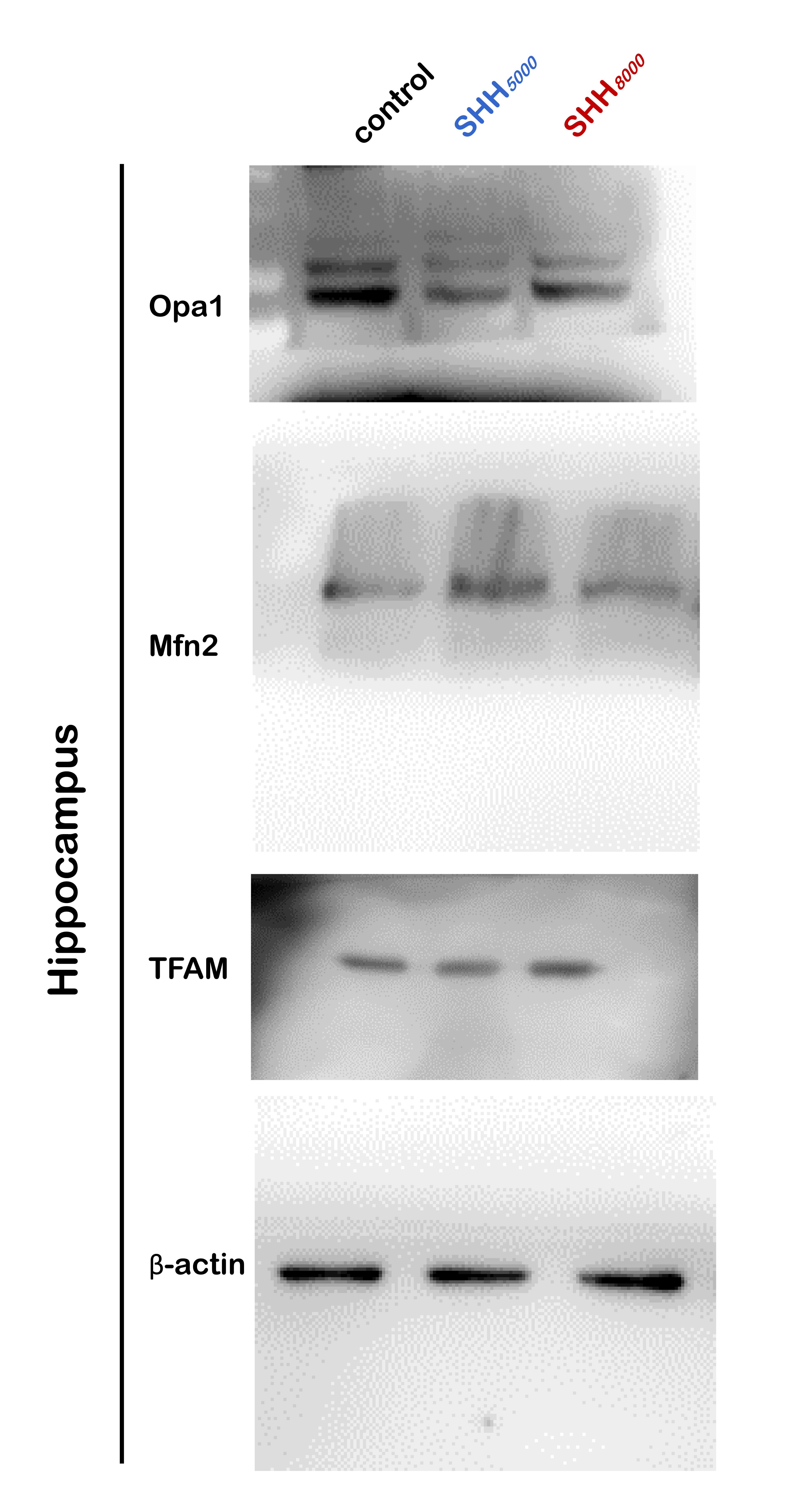

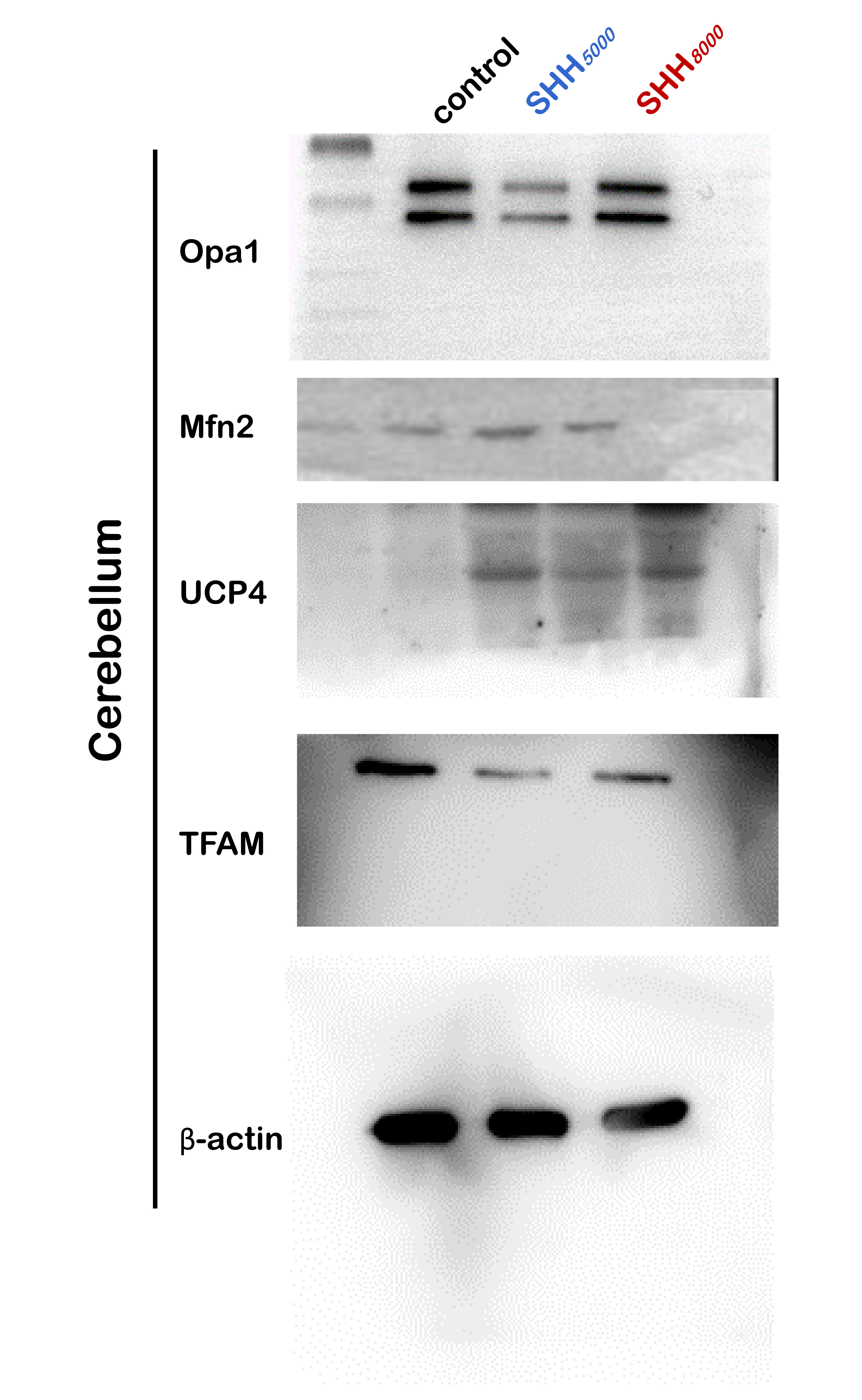


**Additional Information**

**Supplementary videos illustrate the performance from the behavioral tests.**

**Supplementary video 1 with a duration of ten seconds illustrates the performance of the behavioral tests of normal control mice.** The normal mouse walked and moved freely during the first 5 seconds, reared unsupported at 6 - 7 seconds, reared supported at 8 - 9 seconds, and finally sniffed at 10 seconds.

**Supplementary video 2 illustrates that mice present improved locomotor activity and rest more upon mild simulated hypobaric hypoxia at an altitude of 5000 m (**SHH*5000***).** Unexpectedly, after SHH*5000* treatment, the mouse walked and moved quickly (with a much greater velocity) during the first 5 seconds, reared unsupported at 6 - 7 seconds, and rested from the 8th second to the end of the recording.

**Supplementary video 3 illustrates that mice present improved locomotor and exploratory behavior and rest more upon severe simulated hypobaric hypoxia at an altitude of 8000 m (**SHH*8000***).** After SHH*8000* treatment, the mouse walked and moved quickly (with a much greater velocity) during the first 5 seconds, reared unsupported at 6 - 7 seconds, reared supported at 8 - 9 seconds, and finally rested at 10 seconds.
